# Maternal–Infant Bonding and Its Relationships with Maternal Depressive Symptoms, Stress and Anxiety in the Early Postpartum Period in a Polish Sample

**DOI:** 10.3390/ijerph17155427

**Published:** 2020-07-28

**Authors:** Karolina Lutkiewicz, Łucja Bieleninik, Mariusz Cieślak, Mariola Bidzan

**Affiliations:** 1 Institute of Psychology, University of Gdansk, 80-309 Gdańsk, Poland; lubi@norceresearch.no (Ł.B.); cieslak.m@onet.com.pl (M.C.); mariola.bidzan@ug.edu.pl (M.B.); 2 GAMUT—The Grieg Academy Music Therapy Research Centre, NORCE Norwegian Research Centre AS, 5029 Bergen, Norway

**Keywords:** maternal bond, stress, anxiety, postpartum depression

## Abstract

A large body of literature indicates that there is a relationship between maternal psychological well-being and the early maternal–infant bond. However, this relationship is not fully understood, due to the different theoretical frameworks of maternal–infant bonding and different data collections points. Thus, the aim of this study was to examine the relationship between the maternal bond and the maternal psychological state including anxiety, stress, and maternal depressive symptoms. In this cohort study, 150 women who gave birth after 37 weeks of pregnancy completed the following self-reports 1–3 days post-delivery: Socio-demographic questionnaire, Postpartum Bonding Questionnaire (PBQ), Edinburgh Postpartum Depression Scale (EPDS), Postpartum Depression Screening Scale (PDSS), Generalized Anxiety Disorder Assessment (GAD-7), and Parental Stress Scale (PSS). The obtained results showed that the maternal level of stress, anxiety and postnatal depressive symptoms are significantly correlated with the maternal–infant bond in Polish mothers. In addition, regression analysis shows that postpartum depressive symptoms and maternal stress are significantly associated with the maternal–infant bonding process in the early postpartum period. This finding emphasizes the importance of identifying maternal mental state difficulties in the early postpartum period in order to provide interventions to help build healthy maternal–infant bonding.

## 1. Introduction

The process of forming a healthy maternal bond with a child is one of the most significant psychological processes for a mother in the postpartum period and the first year of a child’s life [1], as it affects a child’s survival and healthy future development [2,3]. The concept of bonding first appeared in literature in the mid-1970s and shortly after was popularized by Klaus and Kennel’s (1976) research, who examined the importance of early bonding with a newborn child [4]. Studies have shown evidence that children growing up with the experience of strong maternal bonding are more likely to have better physical, cognitive and psychosocial outcomes as adults. Positive bonding corresponds with a child’s healthy relationships and interactions with other people and determines the parenting of their own children in the future [5,6]. Although the bonding process has been researched for the last few decades, there is a continuing discussion in terms of defining maternal bonding and the analysis of risk factors for impaired bonding measured in different time points among different groups of parents.

Currently, concept analysis of maternal–infant bonding made by Kinsey and Hupcey (2012) [7], shows that in the majority of scientific studies, Maternal–Infant Bonding (MIB) is defined as a process of an emotional tie of a mother to her baby, gradually unfolding in the first year of a child’s life [7,8]. Others consider that the bonding process starts already during pregnancy and continues throughout the child’s life [3]. [For example, research conducted by Van Bussel et al. (2010) showed evidence that a high level of bonding with the baby during pregnancy is associated with high maternal–infant bonding after birth [9]. In contrast, poor bonding between a mother and a child is associated with serious long-term negative impact on a child’s development and mother–infant interactions [1,10].

While the majority of women develop a strong maternal–infant bond, some women may experience disturbances in the process of forming an emotional connection with their child. Studies have reported that impaired maternal–infant bonding occurs in 6 to 41% of mother–infant dyads [11]. The maternal ability to form a bond with a child can be hindered by different factors, such as postpartum depression [12], anxiety [13,14], maternal insecure attachment [15], social support [16,17], preterm birth or a child’s illness [18]. During pregnancy and the postpartum period, women are more vulnerable to experience psychological difficulties [19] such as an increased level of stress, anxiety and depression [20]. The influence of depression symptoms, anxiety and experienced stress on maternal–infant bonding is a complex process that can manifest different reactions and behaviors to the baby.

The literature indicates that postpartum depression is a relatively common mental health problem with a prevalence of between 5% and 60.8% worldwide [21]. A systematic review made by McNamara et al. (2019) showed that prevalence rates of depression ranged from 9–59% [16]. Many studies have presented the association between postpartum depression symptoms and maternal bonding [12,22]. Low mood, lack of joy, low energy, low self-esteem, sleep disorders, mood swings, changes in appetite, fear of injury, serious concerns about the baby, sadness, crying, sense of doubt, difficulty in concentrating, lack of interest in daily activities, thoughts of death and suicide [23] can seriously impact maternal–infant interactions [12], which then contribute to the child’s cognitive and emotional development during infancy and later life [24]. Studies also found an association between maternal depressive symptoms and a lower level of sensitivity to a child [25], including a lack of confidence when taking care of a child [26]. As a consequence, mothers can perceive themselves as less competent as a parent [27] and perception of the child by the mother can also be affected. Negative cognition of a child may distort the responsiveness and interpretations of the child’s behaviors, which in turn affect a mother’s reactions and behaviors toward the baby [28]. 

In terms of the anxiety experienced by mothers in the early postpartum period, research shows that it can range between 8% and 13% [29]. The prevalence rates of anxiety from another study ranged from 25–36% [16]. Differences in prevalence can be explained for example by low participation of mothers with obstetric and neonatal problems, different measurement points and various scales that measure anxiety. Studies showed that postpartum anxiety was connected with an impaired bond, rejection, anger, infant-focused anxieties [30], poorer emotional involvement [13,31] or as an increase in a mother’s awareness and vigilance [28].

Maternal anxiety symptoms can also increase the level of self-reported stress by mothers [27]. Nath et al. (2019) found that the subjective perception of bonding was described as more problematic among mothers with anxiety disorders in comparison to mothers without anxiety disorders [26]. Studies suggest that experienced stress and various stressful life events may have a negative effect on the maternal–infant bonding process [16,32].

Implications on the quality of the maternal–infant bond and the development of a child are consistent in the literature showing that the maternal mental state is connected to the development of maternal–infant bonding. However, there are many discrepancies between studies in terms of methodologies, different measurement points and screening tools [3]. According to our knowledge there has been little research data collected in the first week after birth. Thus, we found it important to examine maternal–infant bonding and the maternal mental state 1–3 days after delivery. The analysis of maternal–infant bonding in different stages of pregnancy and in the first year after delivery can help to understand the development of the bond. Thus, we find it important to know more about the specific factors that may interfere with the quality of maternal–infant bonding in different stages of pregnancy and after birth. Additionally, we measured maternal–infant bonding through the Postpartum Bonding Questionnaire (PBQ) questionnaire, which was used in Poland for the first time. In our study, we also examined anxiety, as the relationship between maternal anxiety and maternal–infant bonding is not widely explored in the literature.

Summing up, we find it crucial to look for risk factors in the early stages of motherhood, to better recognize and identify maternal difficulties and support mothers in developing a healthy bond with their child.

The purpose of this study was to examine the relationship between maternal bonding and the maternal psychological state including anxiety, stress, and depression symptoms in mothers in the early postpartum period (1–3 days after delivery). The following research ultimately explores whether anxiety, stress and depressive symptoms experienced by a mother are associated with maternal–infant bonding in the early postpartum period. 

## 2. Methods

### 2.1. Study Design

This study is a cross-sectional study, which is part of a larger longitudinal study on families after birth (ClinicalTrials.gov ID: NCT04118751). Participation in the project was voluntary. Each participant had the right to refuse to participate in the study or to withdraw from it at any time, for any reason.

In order to be included in the study, women had to deliver a full-term baby (above 37 weeks of gestation, either from a single or multiple pregnancy), be at least 18 years old and sign an informed consent to participate in the project. Mothers with a history of diagnosed mental illness (such as depression, anxiety) and who did not sign the consent form were excluded from the study.

In this study, mothers were enrolled at the Neonatology, Gynecology and Obstetrics ward at the University Clinical Center in Gdańsk with the cooperation of the Department of Psychology at the University of Gdańsk. The project as a whole received ethics approval from the Research Ethics Board at the University of Gdańsk (no 7/2019, date of approval: 29 April 2019). All the participants were recruited between April 2019 and March 2020 by trained assistants in accordance with predetermined eligibility criteria. Initially, eligible women were identified through their medical records. 

First contact with respondents was 24 h after delivery in order to provide information about the project and answer general questions about the study. Mothers were asked to complete anonymous, written self-report questionnaires regarding their subjective level of stress, anxiety, depressive symptoms and perceived bonding with their child, as well as a socio-demographic questionnaire in the early postpartum period before discharge from hospital (collected 1–3 days after delivery).

### 2.2. Outcomes

#### 2.2.1. Primary Outcome

Maternal bonding was measured using the Postpartum Bonding Questionnaire (PBQ, Brockington et al., 2006 [33]; Polish translation, Bieleninik). The self-report scale is a screening tool used to assess the occurrence of disturbances in postpartum bond formation between a mother and child. The method contains 25 statements and uses a 6-point Likert scale for the parents’ answers (each question scores between 0–5, then the points are summed with scores ranging from 0 to 125 (high score = problematic) with a cut-off score of 25) [8]. The questionnaire is also divided into 4 factors. Factor 1 is a general factor and includes 12 questions to identify mothers with a normal bond (a cut-off score of 11; higher scores indicate bonding disorder). Factor 2, rejection and pathological anger is based on 7 questions (a cut-off score of 16) to identify problematic maternal–infant relationships. Factor 3 includes 4 questions (a cut-off score of 9) that aims to identify maternal–infant anxiety and factor 4 includes two questions (a cut-off score of 2) to identify incipient abuse and dangerous behaviors from the mother toward the child [8]. The questionnaire is widely used in clinical practice at the Intensive Care Unit (ICU); however, it has not yet been subject to cultural adaptation in Poland. Łucja Bieleninik obtained approval from the author of the PBQ, Professor Ian Brockington, prior to the translation being done (private correspondence from 29 May 2018 until 27 November 2018). The questionnaire was implemented according to the World Health Organization (WHO)’s recommendation and included: forward translation, expert panel back-translation, pre-testing and cognitive interviewing and final version [34]. The Cronbach α coefficients of reliability in this study group was 0.81 for the full scale.

We included the following secondary outcomes.

1. Maternal depressive symptoms were measured using the Edinburgh Postpartum Depression Scale (EPDS McBride et al., 2014 [35]; Polish development: Bnińska, 1999) [36]. The self-report scale consists of 10 items and is a commonly used screening tool to assess the symptoms of postpartum depression in mothers around the world. It uses a 4-point Likert scale for the maternal answers (each question scores between 0–3, then the points are summed with scores ranging from 0 to 30) [35]. A cut-off score of 10 indicates a strong predictive value for detecting women at risk for developing postpartum depression. The Cronbach α coefficients of reliability in this study group were 0.80 for the full scale.
2. Maternal depressive symptoms were measured using the full Postpartum Depression Screening Scale (PDSS, Polish development: Kossakowska, 2012) [37]. The self-report scale consists of 35 items on a 5-point Likert scale (from 1—definitely I do not agree, up to 5—I strongly agree). Total scores ranged from 35 to 175; the total scores ranging from 35–59 represent normal adjustment, 60–79 significant symptoms of postpartum depression, 80–175 major postpartum depression. The scale helps to establish a pattern of symptoms on 7 dimensions such as: Sleeping/Eating Disturbances (SLP; elevated range ≥14); Anxiety/Insecurity (ANX; elevated range ≥15); Emotional Liability (EML; elevated range ≥15); Mental Confusion (MNT; elevated range ≥14); Loss of Self (LOS; elevated range ≥13); feeling Guilt/Shame (GLT; elevated range ≥13); Suicidal Thoughts (SUI; elevated range ≥6) [37,38] The Cronbach α coefficients of reliability were 0.97 for the full scale and 0.81 for the brief version, ranging from 0.73 to 0.92 for the seven PDSS subscales [37]. The Cronbach α coefficient of reliability in this study group was 0.94 for the full scale.
3. Maternal level of anxiety was measured using the Generalized Anxiety Disorder Assessment (GAD-7, Spitzer et al., 2006) [39]. This is a 7-item self-report questionnaire used as a screening tool and a measure of the severity of generalized anxiety. It differentiates generalized anxiety disorder and comorbid depression as two separate dimensions. The sum of the results ranges from 0 to 21. Cut-off points of 5, 10 and 15 can be interpreted as “mild”, “moderate” and “strong” levels of anxiety. This questionnaire is particularly useful for assessing the severity of symptoms and controlling their change over time. In order to facilitate the assessment of these changes, GAD-7 questions relate to recent symptoms—including the last 2 weeks. The Cronbach α coefficients of reliability were 0.92, while the test–retest reliability intraclass correlation = 0.83 [39]. The Cronbach α coefficient of reliability in this study group was 0.86 for this scale.
4. Maternal level of stress was measured using the Parental Stress Scale (PSS, Berry and Jones, 1995 [40]; Polish translation: Bieleninik), which assesses the stress level associated with parenthood. This is an 18-item self-report questionnaire (each question scores between 1–5). The sum of results ranges from 18 to 90. Higher scores indicate higher levels of stress. The questionnaire considers both positive and negative aspects of parenting [40]. The Cronbach α coefficient of reliability in this study group was 0.82 for this scale.

As presented above, two instruments were used to assess maternal depressive symptoms. In order to receive detailed information about the mental health of mothers in the early postpartum period it was necessary to supplement the EPDS information with data from the second instrument PDSS. While EPDS provides information about major postpartum depression, PDSS consists of 7 additional dimensions such as: sleeping/eating disturbances; anxiety/insecurity; emotional lability; mental confusion; loss of self; feeling guilt/shame; suicidal thoughts.

Other measures 

The following socio-demographical records were collected from mothers:

1. Maternal age (years);
2. Marital status (single/married/living together, not married/divorced/separated/widow);
3. Education level (primary or elementary or less/secondary school, but not completed/secondary school graduate/university, but not completed/university degree (bachelor or equivalent)/university degree (master or equivalent)/university degree (PhD or equivalent));
4. Work status (employed/paid maternity leave/neither);
5. Number of children in the household (including the child participating in this study);
6. History of anxiety/depression (yes/no);
7. History of using of antidepressants (yes/no).

Information about the children was collected from hospital records and included: gender (male/female), birth weight (grams), delivery route (vaginal/cesarean), final APGAR score (A-appearance (skin color), P-pulse (heart rate), G-grimace (reflex irritability), A-activity (muscle tone), R-respiration). All data were obtained in paper datasets and extracted into Microsoft Excel.

#### 2.2.2. Statistical Methods Plan

Socio-demographic and clinical properties are characterized by descriptive methods (mean (SD), median (range), *n* (%)). In order to examine the normality of the distribution of the analyzed parameters, the Kurtosis–Skewness test (K-S test) and basic statistics were used for quantitative variables included in the group. Distributions of tested variables were similar to normal distributions, which were confirmed by the results of K–S tests and analysis of the values of each available kurtosis coefficient and skewness for the tested compounds.

## 3. Results

### 3.1. The Characteristic of Study Group

The study group consisted of 150 women who gave birth after 37 weeks of gestation. The participation rate was 100%, including 150 women (M = 30.71; SD = 4.03). The mothers’ average age was *M* = 30.71 years old (*SD* = 4.03). In total, 78% of participants were in a relationship (partner or spouse), and 78% declared to have higher education. Further, 50% of the women had one child, 35% two. The average weight of a newborn child was *M =* 3481.9 (*SD =* 450). The average result on the APGAR scale was *M =* 9.93 (*SD =* 0.33). A detailed characteristic of the group is presented in the Table 1.

### 3.2. Maternal Bonding Scores

The average intensity of postpartum bonding was measured subsequently in the group (Table 2). The average level of emotional bonding is 8.57 points and corresponds to the entire scale. This is a very low result indicating a very good emotional relationship between the mother and the newborn child. Results analysis of the average values for four individual factors indicates a low intensity of the examined variables, which indicates the absence of disorders in the examined mothers.

### 3.3. Maternal Stress, Anxiety and Depression Scores 

The average intensity of independent variables was measured subsequently in the group (Table 3). The obtained results indicate a low level of postnatal depression in the examined women. The obtained result is below 10 points. The result obtained on the PDSS scale indicates the norm. The result is in the range of 35–59 points. The average result obtained in the GAD-7 questionnaire indicates a moderate severity of anxiety symptoms in the examined women. In the case of stress, the examined women show a low level (32.79 points).

Extending the analysis of the results obtained in the case of EPDS-27 (18%) women obtained a score higher than 10 points, which indicates a probable depression. In PDSS-45 (30%) women obtained a score higher than 59, which may also indicate postpartum depression. In the case of GAD, 61 (41%) mothers scored below 10 points, 63 (42%) below 15 points, and 26 (17%) above 15. There are no cut-off points in PSS, the lower the score, the less stress.

### 3.4. Correlations between Maternal–Infant Bonding, Maternal Level of Stress, Anxiety and Postnatal Depressive Symptoms

The next phase of the analysis aimed to indicate the initial correlations between maternal–infant bonding and postnatal depressive symptoms, stress and anxiety in the tested group. The results are presented in the tables below (Table 4). The obtained results show a positive and moderate correlations between the overall result of maternal bonding and depressive symptoms, stress and anxiety. There were also positive, strong correlations between maternal bonding and experienced stress by mothers and postnatal depressive symptoms (PDSS). The correlations analysis showed a positive, moderate correlation between the maternal bonding and depressive symptoms (EPDS). Rather weak, positive correlation appeared between maternal–infant bonding and symptoms of anxiety.

The detailed corrections, including four factors of PBQ scale, are presented below (Table 4). 

### 3.5. Regression Analysis of Maternal Bonding with Maternal Stress, Anxiety and Postnatal Depressive Symptoms

In the final step of the results analysis a linear regression was performed on the whole group of participants to examine the connection between maternal–infant bonding and maternal mental health. The maternal–infant bonding was the explained variable and symptoms of depression, anxiety and stress were the explanatory variables. The regression model turned out to be fitted to the data F (4, 136) = 30.410, *p* <0.01. The linear regression analysis indicated the important role of the overall scores in depressive symptoms (EPDS) and experience of stress (PSS) among participants. In both cases, based on the value of the B factor, it can be concluded that the higher the EPDS score in the examined group and the higher the PSS level, the higher the results in PBQ (more problematic bond between mother and her child). The regression model explains 46% of the variability of the dependent variable (R^2^ = 0.464). When predicting the PBQ level on the basis of the constructed regression model, the value of the standard error of estimation was 5.04. The obtained results are presented in Table 5.

## 4. Discussion

The first aim of this study was to examine whether maternal mental state, including anxiety, depressive symptoms and experience of stress, is related to maternal–infant bonding in the early postpartum period. 

With respect to the research question, it was found that there were positive and strong associations between maternal–infant bonding with postpartum depressive symptoms and maternal level of stress. The findings are in line with the previous studies that demonstrated the association between postpartum depression symptoms and maternal bonding during the antenatal and early postpartum period [12,22,41]. Many studies researched the connection between maternal bonding and postpartum depressive symptoms at 6 weeks after delivery or later. For example, Nakano et al. (2019) found that postpartum depression measured 1 month after delivery is strongly associated with impaired maternal bonding measured 12 weeks after the delivery [3]. This finding suggests that maternal depressive symptoms measured in the early postpartum period, such as 1–3 days after delivery, could have an effect on future maternal bonding and the relationship with a child. This raises the importance of pre-screening mothers for maternal depressive symptoms after delivery and then continuing to monitor their mental state [3]. A systematic review conducted by McNamara et al. (2019) found that 15 of 19 research studies showed the evidence that postpartum depression is associated with maternal–infant bonding [16]. 

In this study, we also found a strong correlation between stress and maternal–infant bonding. This result is confirmed by other studies, suggesting that stress may have a negative effect on the maternal–infant bonding process [16,32]. Contrary to these results, other studies found that mothers, while experiencing high levels of stress, presented a stronger bond with the baby and higher levels of responsiveness and sensitivity to the baby’s needs [32,42]. 

According to a systematic review conducted by McNamara et al. (2019), only two reports examined the relationship between anxiety and maternal–infant bonding [16]. Other evidence from research studies suggested that anxiety is another factor, which can significantly impair maternal–infant bonding [13]. In this study, the correlation between maternal–infant bonding and anxiety symptoms was positive and weak. Figueiredo and Costa (2010) showed that anxiety is related to poorer maternal–infant bonding, which was demonstrated by negative emotions toward the baby and lower levels of emotional involvement while taking care of the baby [31]. In another study, the subjective perception of bonding was described as more negative among mothers with anxiety disorders in comparison to mothers without anxiety disorders [26]. A similar result was obtained by Tietz et al. (2014), where mothers reported poorer bonding with their babies while having postpartum anxiety disorder [43]. 

The findings from this study are supportive to the claim that maternal mental state can be negatively related with the ongoing process of maternal–infant bonding formation in the early postpartum period. The study results have important clinical implications by raising the importance for pre-screening maternal mental state after delivery to provide needed help and increase supportive resources for women to give an opportunity for development of a strong and healthy bond between a mother and her baby. This study adds to the literature showing that not only postpartum depressive symptoms, but also maternal stress are risk factors toward a negative relationship between a mother and her child. This is a key issue since problematic maternal–infant bonding is crucial for the future child’s developmental outcomes [2,3]. 

### 4.1. Limitations

There were several limitations that need to be acknowledged in this report. First, all the information about maternal mental state, including maternal–infant bonding, maternal depressive symptoms, stress and anxiety, were assessed using self-reports. In studies that use self-report measurement tools, there is a higher risk of bias compared to clinically observed outcomes in unblinded designs. Second, it is also important to interpret the results carefully, as in this study the maternal–infant bonding was good. Third, outcomes were limited to one time point (maternal–infant bonding and maternal mental state were measured only during the first three days after delivery). Having follow-up points of maternal–infant bonding and its predictors could show the trajectory over time as well as establish whether there is a risk to sustain the obtained associations after discharge from hospital. Fourth, it was not possible to show causality between the variables. Fifth, the obstetric background, including negative experiences from previous pregnancies or mental health history, was not verified in the study, hence it is recommended to take this variable into account in subsequent studies. Without a doubt, there is a call to evaluate psychometric properties of a translated Polish version of the PBQ in mothers, which the authors plan to undertake in the next step. These findings cannot be extrapolated to all patients since the project was conducted in one unit. 

### 4.2. Implication for Further Research

Despite these promising results, questions remain. For further research it would be valuable to gather more specific information about how exactly stress and coping strategies with stress are related to maternal–infant bonding. There is abundant room for further progress in determining other risk factors that may affect maternal bonding with a child such as social support, parental relationship quality, maternal body image [16], and optimism and resilience [44]. In general, understanding how maternal mental state is related to a mother’s perceptions of bonding and her behavior towards a child may help to provide various interventions for mothers in order to prevent potential negative outcomes [45]. Future investigations should track the trajectory of bonding over time. Taken all together, future studies on the current topic are therefore recommended.

## 5. Conclusions

The present results are significant in at least two major respects. One of the issues that emerge from these findings is that there are significant associations between bonding and maternal level of stress, anxiety and postnatal depression in Polish mothers. Second, the present study raises that postpartum depressive symptoms and maternal stress are significant risk factors for the formation of a positive relationship between a mother and her child in the early postpartum period. Due to all the aforementioned limitations, these results therefore need to be interpreted with caution.

## Figures and Tables

**Table 1:** Descriptive characteristics of the study group (*n* = 150). APGAR: (A-appearance (skin color), P-pulse (heart rate), G-grimace (reflex irritability), A-activity (muscle tone), R-respiration).

**Marital Status**	***n* (%)**
Single	2 (1.3%)
Married	117 (78%)
Partnership	28 (18.7%)
Divorced	1 (0.7%)
Separated	1 (0.7%)
Widow	0 (0%)
Other	1 (0.7%)
**Educational Background**	
Elementary education or less	0 (0%)
High school (but not completed)	3 (2%)
High school	26 (17.3%)
University (but not completed)	4 (2.7%)
Higher education (bachelor’s degree)	26 (17.3%)
Higher education (master)	88 (58.7%)
PhD	3 (2%)
**Type of Employment**	
Full-time employment	103 (68.7%)
Part-time employment	6 (4%)
Owner of their own company	14 (9.3%)
Full-time studies	3 (2%)
External studies	2 (1.3%)
Staying home mother	16 (10.7%)
Unemployed	2 (1.3%)
Unemployed due to poor health or disability	0 (0%)
Other	4 (2.7%)
**Number of Children in the Family**	
One	75 (50%)
Two	54 (36%)
Three	16 (10.7%)
Four	4 (2.7%)
Five	1 (0.7%)
**Type of Birth**	
Vaginal birth	76 (50.7%)
Cesarean birth	74 (49.3%)
**Sex of a Child**	
Boy	79 (52.7%)
Girl	71 (47.3%)
**APGAR**	
8 points	3 (2%)
9 points	5 (3.3%)
10 points	142 (94.7%)

**Table 2:** Descriptive characteristics of maternal–infant bonding (PBQ) of the study group (*n* = 150).

PBQ	Median	SD	Minimum	Maximum
General factor	3.91	3.64	0	16
Rejection and pathological anger	1.67	2.07	0	10
Anxiety about the infant	2.90	2.17	0	11
Incipient abuse	0.03	0.01	0	2
Average scores	8.57	6.81	0	30

Annotation: PBQ, Postpartum Bonding Questionnaire; SD, Standard Deviation; M, Median.

**Table 3:** Descriptive characteristics of EPDS, PDSS, GAD, PSS (*n* = 150).

Item	Median	SD	Minimum	Maximum
EPDS	6.87	4.13	0	22
PDSS	55.19	16.84	35	120
Sleeping/eating disturbances	10.07	3.75	5	21
Anxiety/insecurity	9.04	2.77	5	19
Emotional liability	8.80	3.61	5	22
Mental confusion	7.99	3.39	5	19
Loss of self	6.49	2.25	5	18
Feeling guilt/shame	7.30	3.06	5	21
Suicidal thoughts	5.47	1.26	5	12
GAD	12.20	4.26	7	21
PSS	32.79	7.63	18	53

Annotation: EPDS, Edinburgh Postnatal Depression Scale; GAD-7, Generalized Anxiety Disorder Assessment; PBQ, Postpartum Bonding Questionnaire; PDSS, the Postpartum Depression Screening Scale; PSS, Parental Stress Scale; SD, Standard Deviation; M, Median.

**Table 4:** Detailed correlation results of PBQ subscales with depression, anxiety and stress.

Variable	EPDS	PDSS	GAD-7	PSS
PBQ	0.456 **	0.512 **	0.316 **	0.642 **
Rejection and pathological anger	0.437 **	0.494 **	0.345 **	0.494 **
Anxiety about the infant	0.305 **	0.367 **	0.194 *	0.367 **
Incipient abuse	0.417 **	0.381 **	0.266 **	0.381 **
Average scores	−0.011	0.125	−0.030	0.125

** *p* < 0.01, * *p* < 0.05. Annotation: EPDS, Edinburgh Postnatal Depression Scale; GAD-7, Generalized Anxiety Disorder Assessment; PBQ, Postpartum Bonding Questionnaire; PDSS, Postpartum Depression Screening Scale; PSS, Parental Stress Scale.

**Table 5:** Significance of the standardized regression coefficients describing the relationship between PBQ scores and depressive symptoms, anxiety and stress.

Variable	β	*B*	SE *B*	t	*p*	VIF
EPDS	0.244	0.411	0.168	2.444	0.016	2.120
PDSS	0.119	0.048	0.036	1.347	0.180	1.983
GAD	−0.080	−0.133	0.151	−0.879	0.381	2.086
PSS	0.515	0.458	0.067	6.845	0,000	1.435
Constant value		−10.366	2.170	−4.777	0.000	

R = 0,693; R^2^ = 0,464. Annotation: EPDS, Edinburgh Postnatal Depression Scale; GAD-7, Generalized Anxiety Disorder Assessment; PBQ, Postpartum Bonding Questionnaire; PDSS, Postpartum Depression Screening Scale; PSS, Parental Stress Scale; the standardized beta (β); the unstandardized beta (B); the standard error for the unstandardized beta (SE B); the t test statistic (t); the probability value (*p*); R, coefficient of determination; VIF, variance inflation factor.
